# Nanodiamond phantoms mimicking human liver: perspective to calibration of T1 relaxation time in magnetic resonance imaging

**DOI:** 10.1038/s41598-020-63581-9

**Published:** 2020-04-15

**Authors:** Anna Sękowska, Daria Majchrowicz, Agnieszka Sabisz, Mateusz Ficek, Barbara Bułło-Piontecka, Monika Kosowska, Lina Jing, Robert Bogdanowicz, Małgorzata Szczerska

**Affiliations:** 1 Department of Medical Physics, Pomeranian Medical University, 12 Ku Słońcu Street, 71-080 Szczecin, Poland; 2 Department of Metrology and Optoelectronics, Faculty of Electronics, Telecommunications and Informatics, Gdańsk University of Technology, 11/12 Narutowicza Street, 80-233 Gdańsk, Poland; 3 SUPA School of Physics and Astronomy, University of St Andrews, North Haugh, KY16 9SS St Andrews, UK; 4 2-nd Department of Radiology, Medical University of Gdansk, 3a Marii Skłodowskiej-Curie Street, 80-210 Gdansk, Poland; 5 Department of Nephrology, Transplantology and Internal Diseases, Faculty of Medicine, Medical University of Gdańsk, 3a Marii Skłodowskiej-Curie Street, 80-210 Gdansk, Poland; 6 Department of Radiology, Beijing Tiantan Hospital, Capital Medical University, 119 West Fourth Ring South Road, Fengtai District, 100070 Beijing, China

**Keywords:** Medical research, Preclinical research

## Abstract

Phantoms of biological tissues are materials that mimic the properties of real tissues. This study shows the development of phantoms with nanodiamond particles for calibration of T1 relaxation time in magnetic resonance imaging. Magnetic resonance imaging (MRI) is a commonly used and non-invasive method of detecting pathological changes inside the human body. Nevertheless, before a new MRI device is approved for use, it is necessary to calibrate it properly and to check its technical parameters. In this article, we present phantoms of tissue with diamond nanoparticles dedicated to magnetic resonance calibration. The method of producing phantoms has been described. As a result of our research, we obtained phantoms that were characterized by the relaxation time T1 the same as the relaxation time of the human tissue T1 = 810.5 ms. Furthermore, the use of diamond nanoparticles in phantoms allowed us to tune the T1 value of the phantoms which open the way to elaborated phantoms of other tissues in the future.

## Introduction

Magnetic resonance imaging (MRI), is a non-invasive technique for creating detailed images of the human body. This method allows for obtaining high-resolution cross-sectional images of tissue in any surfaces. Therefore now is a widely available, powerful instrument for non-invasive imaging of soft tissues better then X-rays, computerized tomography scans or ultrasound^1–3^. Chronic liver diseases represent an important global health problem.

MRI is a technique which plays a significant role in diagnosis of different liver disorders. The MRI investigation can show and confirm diffuse liver damage due to metabolic diseases or cirrhosis. Furthermore, MRI plays an important role in differentiation in primary hepatic malignancies or metastatic diseases^4^. The parameters characteristic for a sample investigated by the means of MRI are longitudinal relaxation time T1 and transverse relaxation time T2, which directly depend on the biophysical structure of the test substance and the biochemical environment^5^. The study of relaxation times and their dependence on the magnetic field, temperature, pH, etc. provides valuable information on the structure and molecular dynamics of the substance. A significant amount of biological tissue phantoms used for an MRI scanner calibration is created to examine the scanner’s technical parameters^6–13^. Phantoms used to calibrate relaxation times are scarce. The few phantoms dedicated to the calibration of relaxation times are mainly oil based or water based (e.g. aqueous solutions of inorganic acid salts^12,14–17^). Due to their liquid state, the calibration process is more time-consuming because the liquid must stabilize before the measurement can start. Relaxation agents such as gadolinium trichloride, which were found to enable adjustment of T_1_ relaxation time, are toxic, therefore their use for biomedical applications is excluded^18^.

In this paper, we report the development and magnetic characteristics of liver phantoms for the calibration of an MRI scanner. They were produced on the basis of agar and carrageen, and diamond nanoparticles. The obtained phantoms with the addition of diamond nanoparticles (size of 4–5 nm) had relaxation time T_1_ very similar to the selected human body tissue - liver.

## Experimental – Phantom characteristics

The MRI imaging was performed using phantoms containing nanodiamond particles fabricated by detonation method (DND) and purified by Ray Techniques Ltd. (Israel). The utilized nanodiamond particles have hydroxyl-terminated surface revealing hydrophilic nature as pointed out by Ray Techniques Ltd. (RT-DND-L). The RT-DND particles are monocrystalline, poly-dispersed with average size of 4–5 nm. The dimethyl sulfoxide (DMSO) was applied here as a dispersant of RT-DND particles in agreement with deagglomeration procedures and DND-DMSO interactions reported by Shenderova *et al*.^19,20^.

The RT-DND particles were dispersed in DMSO using ultrasound Bandelin Sonopuls HD 2200 sonicator (Berlin, Germany), working in a pulse mode to avoid overheating of the liquids^21^. The suspension was prepared by 5-min-long high-power ultrasound sonication which disintegrates partially the ND aggregates, allowing for marginal effect on surface modification. The 0.1  wt.% RT-DND concentration in DMSO was applied. The process resulted in colloidally stable suspension with average aggregate size of 70–80 nm, being in agreement with previous data shown by Shenderova *et al*.^19^. The particle sizes were measured with dynamic light scattering (DLS) using a Malvern Zetasizer Nano ZS instrument (UK). The content of RT-DND:DMSO suspension in prepared phantoms DNP-0, DNP-8, DNP-10, DNP-12 was set to 0%, 8%, 10% and 12%, respectively.

**Figure 1:**
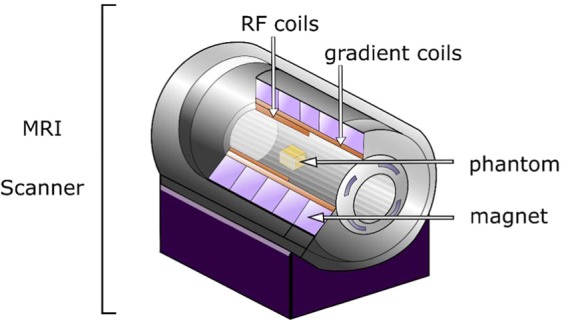
Visualization of the experiment.

Due to the fact that the prepared phantoms are to be used in calibration of magnetic resonance scanner (Fig. 1), two types of measurements were carried out: T1 and T2 relaxation time measurements. T1 measurement was performed in order to verify whether nanodiamond particles can be used as T1 modifiers in MRI phantoms. T2 measurement was carried out to check the nanodiamonds influence on obtained T2 values.

The first type of measurement was the measurement of relaxation time T1 using the Inversion Recovery method. A series of scans was obtained for four different inversion times TI: 200, 400, 800, 1200 [ms], at four different depths. 20 measurements were taken for each sample from a given group (5 on each of 4 scans). Based on the acquired scans T1-maps for each slice were obtained with the use of MRmap program (Mountain Rescue, UK). Moreover, the investigated phantoms were measured with the use of a multi-echo spin-echo sequence. A series of phantom scans for six moments of time 1 TE (13 ms), 2TE, 4TE, 5TE, and 6TE on ten different depth levels was obtained. The maps of relaxation time T_2_ for each slice were achieved by the means of RadiAnt software (Medixant, Poland). For the obtained maps, measurements of relaxation time T2 were carried out at different phantom depths, each time five measurement areas located centrally on one phantom layer were tested. For each phantom, 25 measurements were taken. MRI imaging was performed at the Gdansk University of Medicine by the trained medical physics, supervised by Medical Doctor of Radiology.

## Result

**Figure 2:**
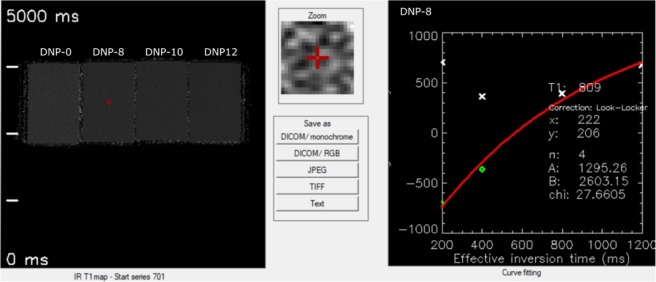
T1-map of phantoms with nanodiamond RT-DND-L suspension and DMSO and T_1_ Recovery curve for phantom DNP-8.

The first type of measurement, which was made for phantoms with the addition of detonation nanodiamonds and DMSO, was the measurement of relaxation time T1 using the Inversion Recovery method. Exemplary T1-map of phantoms with nanodiamond RT-DND-L suspension and DMSO and T1 Recovery curve for phantom DNP-8 are presented in Fig. 2.

**Figure 3:**
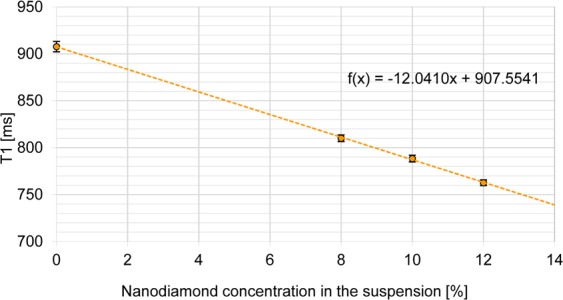
Relaxation time T1 of the produced phantoms as a function of the nanodiamond concentration in the used suspension.

The results of relaxation time T1 measurements are presented in Fig. 3. A linear regression curve was adopted (model of linear changes of T1 relaxation time with the change of suspension content in the phantom) and its coefficient of determination was calculated, R^2^ = 0.9997.

**Figure 4:**
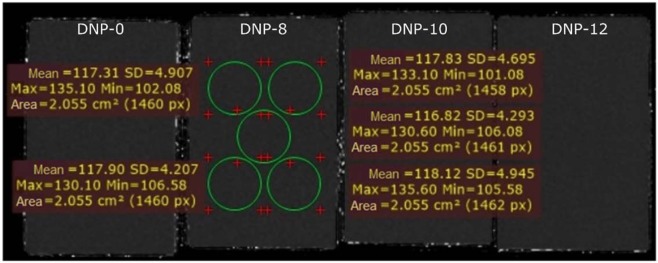
T2-map of phantoms with nanodiamond RT-DND-L suspension and DMSO, and T2 measurements for phantom NDS-8.

On the basis of the relaxation time T1 measurements, we can see that the dependence of relaxation time on the content of nanoparticles is linear. This allows a simple way to make phantoms of specific parameters. The correlation coefficient R^2^ is almost equal to one, which indicates that the adopted mathematical model nearly ideally describes the measurement data. The appropriate adjustment of nanodiamond concentration in the examined range will allow production of phantoms reflecting the properties of the liver. Next, the relaxation time T2 was measured. The obtained results are presented in Fig. 4.

**Figure 5:**
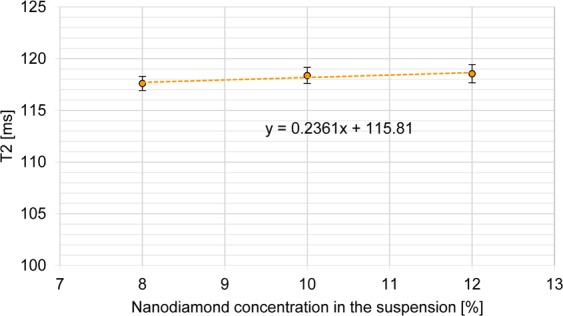
Relaxation time T2 of the produced phantoms as a function of the concentration of the used suspension.

The results of relaxation time T2 measurements are presented in Fig. 5. The correlation coefficient of the linear fit is equal to R^2^ = 0.8838.

Phantoms with the addition of nanodiamond suspension provide stable values of T2, similar to the pure agar-carageen phantom (117.92 ms ~ 118.55 ms). This suggests that the value of relaxation time T2 can be tuned by proper adjustment of agar content^17^. We obtained phantoms which were characterized by the relaxation time T1 = 810.1 ms the same as the relaxation time of the human liver T1 liver = 810.5 ms^22^. The relative error of the relaxation time T1 of the liver and the produced phantom is 0.049%. The relaxation time T2 of the studied phantom (117.6 ms) significantly differs from the characteristic value of the liver tissue (38.0 ms). However, an adequate adjustment of the agar concentration will also allow the adjustment of T2 relaxation time, which is the subject of further research. Taking into consideration obtained result we can say that we elaborated a solid phantom mimicking the human liver specific in terms of its T1 relaxation time. We obtained the value of T1 at the same level as the one reported in the medical database and even though the T2 is not yet properly tailored now, we expect it to be achievable by change of agar content in the phantom.

## Discussion

Until now, a few groups have reported production of MRI phantoms. For example, Bazrafshan *et al*. reported a liver polyacrylamide MRI gel phantom mimicking the T1 and T2 of liver up to 75 °C making it possible to be used in thermal therapy^23^. However, T1 values measured at different temperatures differ from the tissue values by around 10–15%. Kato *et al*. presented MRI phantoms mimicking times T1 and T2 for desired human tissue with an accuracy of 1 ms^18^. The preparation procedure requires many ingredients though, making it relatively complicated. Brzozowski *et al*. proposed MRI phantoms with gellan gum-based gels instead of agar, which were doped with superparamagnetic iron nanoparticles as T2 modifier and either MnCl_2_ or GdCl_3_ as T1 modifiers^24^. The presented mathematical model showed the relation between a concentration of contrast agent with adj. R^2^ > 0.97 for longitudinal relaxation rates, and adj. R^2^ > 0.87 for transverse relaxation rates.

The main objective of this study was to present the method for producing phantoms of tissue with the use of nanodiamond particles for calibration of magnetic resonance scanner. We showed the linear relation between the value of T1 and concentration of nanodiamonds allowing production of magnetic phantoms of desired tissue. The goal was achieved and the T1 parameter of our phantom is the same as a T1 value for liver tissue. The changes in T1 with the change of nanodiamond concentration in the used solution is described by correlation coefficient R^2^ = 0.9997 (adj. R^2^ = 0.9996). This gives us the possibility to produce the phantoms of other tissue. The study needs further investigation regarding the tailoring of T2 value. However, it was shown that this parameter remains stable regardless of nanodiamonds content, meaning that T1 modification by nanodiamonds does not affect the T2. *On*e of the basic limitations of the study was the availability of only one MRI scanner with a magnetic field strength of 3.0 T. It would be advisable to study the relaxation times of the prepared phantoms for MRI scanners with different magnetic field strengths in order to verify the results.

## Conclusions

While phantoms mimicking selected properties of tissues are used in testing and verifying measurement devices, there is still a lack of the phantoms for magnetic resonance calibration. Therefore, we developed a new group of phantoms utilizing diamond nanoparticles, which are dedicated for MRI calibration. We found that the relaxation time T1 of these phantoms is easily tunable because of the linear relationship between its value and concentration of the nanodiamonds. This unique solution provides a possibility to tailor the T1 parameter to desired value in a simple, cost-effective and fast (few hours) way. Furthermore, the developed phantoms are produced in a gel form. Commonly produced phantoms in a liquid form show many limitations: not only is the time of their use relatively short, but also obtaining the multi-layer systems is impossible. The examination procedure is also unnecessarily prolonged due to the requirement of liquid stabilization. The proposed approach solves the aforementioned problems, while keeping the simplicity of preparation procedure.

In this paper, phantoms for an MRI scanner calibration with nanodiamonds were presented. RT-DND-L detonation nanodiamonds were used during the investigation. Measurements of their magnetic properties were made in order to verify the possibility of their use for the calibration of magnetic resonance scanner. The addition of the suspension did not significantly affect the change in the relaxation time T_2_ (this parameter was more stable though) and the dependence of time T1 on the suspension concentration turned out to be linear. The obtained phantoms with the addition of diamond nanoparticles with an average size of 4–5 nm were characterized by the relaxation time T1 which is very similar to the selected tissue of a human body - liver. The addition of the suspension has a significant effect on the change of relaxation times T1 and T2, and the relationship in the case of relaxation time T2 does not show linearity. However, the analysis of the measurements of the relaxation time T2 shows that the addition of suspension affected the change in the measured value to some extent. The proper tailoring of the T2 parameter is the subject of our further investigations.

Quantitative MRI is now one of the top methods in radiology. There are growing number of articles and hence acquisition protocols in clinical studies considering T1 measurements. The use of such phantoms will allow for verification of accuracy of results by different MRI. Such phantom can help to optimise the instrumental precision and support quality control. Due to their solid form, the calibration process will be shortened. In addition, these phantoms do not contain any toxic compounds in contrast to frequently used T1 modifier - gadolinium chloride. Furthermore, our investigation open the new possibility to create the solid phantoms of other tissues and organs for MRI calibration in the future, which has stared to be a standard in other methods of biological samples imaging like optical imaging^25,26^.

## Methods

### Nanodiamond preparation

The method of phantoms production was developed on the basis of the technique using agar media for microbiological tests. The bases of the produced phantoms are agar-carrageen gels. The mechanical properties of the phantoms mainly depend on carrageen, and changes in the relaxation time T2 are dependent on agar. The studies determined that carrageenan concentration should not exceed 2% due to technological difficulties. The composition of the target phantoms for a magnetic resonance scanner calibration was enriched by the addition of nanodiamond suspension. Nanodiamonds were added to the agar-carrageen gel to modify the relaxation time T1. The solution base (the dispersing phase), is dimethyl sulfoxide (DMSO). In this article, the phantoms with the addition of a 0.10% suspension prepared on the basis of RT-DND-L nanodiamond powder (Ray Techniques Ltd Israel) containing 8% to 12% of the nanoparticles with an average size of 4–5 nm were performed.

### Phantom fabrication

Distilled water, agar (1.413%) and carrageenan (2%) were used to produce a group of phantoms with nanoparticles of a certain type, differing in the concentration of the suspension. In order to fully distribute the agar and carrageenan, the components were heated to boiling, stirred and put into the autoclave for about 45 minutes, with the inside temperature of 126 °C. The gel base of phantoms was poured into the prepared nanodiamond suspensions. The contents of the individual beakers were then thoroughly mixed and subjected to the autoclave. Then the samples were poured into molds and placed under cover to congeal.

### MRI setup

Magnetic resonance imaging was performed with the Philips Healthcare Achivea 3.0T-TX using a 32-channel coil dedicated for the examination of the heart. MultiTransmit technology used in this system eliminates dielectric shading by applying simultaneous transmission from multiple RF signal sources, ensuring optimal homogeneity and consistency of the image and faster scanning rate [19]. Measurements of relaxation time T1 were performed using the Inversion Recovery (IR) method. The measurement parameters were as follows: TR pulse repetition time was 1600 ms, TE echo time per 15 ms, TI inversion times: 200, 400, 800, 1200 ms, voxel size 0.9375 × 0.9375 × 3 mm. Measurements of relaxation time T2 were carried out using the multi-echo method using the spin echo technique. The measurement parameters were as follows: the pulse repetition period was set to 2000 ms, TE 13 ms echo time, voxel size 0.75 × 0.75 × 2.5 mm.
